# Masticatory Force in Relation with Age in Subjects with Full Permanent Dentition: A Cross-Sectional Study

**DOI:** 10.3390/healthcare9060700

**Published:** 2021-06-09

**Authors:** Ottavia Poli, Licia Manzon, Tarcisio Niglio, Evaristo Ettorre, Iole Vozza

**Affiliations:** 1 Dental School, “Sapienza” University, 00185 Rome, Italy; ottavia.poli@uniroma1.it (O.P.); licia.manzon@uniroma1.it (L.M.); 2 Istituto Superiore di Sanità, 00161 Rome, Italy; tarcisio.niglio@iss.it; 3 Department of Cardiovascular, Respiratory, Nephrological, Anesthetic and Geriatric Sciences, “Sapienza” University, 00185 Rome, Italy; evaristo.ettorre@uniroma1.it

**Keywords:** dentition, permanent, masticatory muscles, bite force

## Abstract

Masticatory performance is directly correlated with masticatory muscle work to grind and cut the food. Chewing efficacy is decisive to eating a variety of foods needed maintain general health status at all ages. Older people have oral problems that get worse with age. Elders have more pathologies such as periodontal diseases, caries, tooth loss and inadequate dental prostheses than younger subjects. Objectives: to investigate the correlation between masticatory bite force (MBF) and body mass index (BMI) vs. aging and sex. Methods: This study was performed on 426 subjects (213 females plus 213 male) assigned into five different groups by age. Group “A” aged from 20 to 35 years; group “B” aged 45–59 years; group “C” aged 60–69 years; group “D” aged 70–79 years; and group “E” aged more than 79 years. Results: There were not statistically significant differences in right-side MBF versus left-side MBF. The differences between sex were statistically significant with a stronger bite in males than females (*p* < 0.05). At the same time, younger subjects had a stronger bite than elders (*p* < 0.05). In group “E”, more corpulent subjects (BMI > 25) had an MBF higher than less corpulent subjects (BMI < 25, *p* < 0.05). The analysis of mean MBF showed a statistically significant difference within all groups stratified by BMI with mean values inversely proportional with age (*p* < 0.001). Conclusion: The results in our study confirm data from many scientific papers. The importance of the present paper was to correlate data between and within a large sample with a wide range of ages. Our sample subjects had a 31%–33% decrease in MBF from group “A” to group “E” group, but they all had full permanent dentation and they preserved a valid MBF.

## 1. Introduction

Chewing and swallowing processes are critical for normal food intake. The masticatory process is influenced by many factors such as the number of healthy residual teeth, the number of occlusal contacts, the flow rate of salivary, the periodontal status, the jaw muscle activity and bite force [1]. Public health expenditure could be reduced and quality of life could be improved if all these factors were maintained efficiently [1,2,3,4].

Older people have oral problems that get worse with age such as periodontal diseases, caries, tooth loss and inadequate dental prostheses [5]. Oral status influences people in food selection and nutritional well-being. Indeed, elders prefer soft food with low-density proteins because it is easy to chew. Unfortunately, these foods frequently promote increasing body weight with the consequent risk of obesity, diabetes, hypertension and cardiovascular diseases [5,6]. Nutritional status is also very important in elder mental health, physical and cognitive functions, as well as mobility and independence [7]. People who have lost canines also have a reduced masticatory performance, even if equipped with mobile dentures. Moreover, the number of posterior opposing teeth and bite force are determinant for masticatory performance [1,5,8]. Considering geriatric patients, there are even more factors involved in determining mastication efficiency. Indeed, with age, there is a paraphysiologic decrease of muscle mass and strength that might be associated with pathological sarcopenia, which is associated with a worse neuromuscular control, such as peripherical neuropathy that is often found in chronic diseases like diabetes [9,10,11,12]. To sum it up, masticatory performance can be seen as a factor and consequences of frailty, with decreased physiological reserves and increased vulnerability [13]. Masticatory performance is directly correlated with masticatory muscles’ work in grinding and cutting food. The measurement of maximum voluntary bite force (MBF) reveals masticatory system activity, and it is correlated with many factors such as dentition status, dental prosthesis and temporomandibular disorders [14]. Many patients adapt their loss of masticatory capability by chewing food for a longer time or using more chewing cycles [15]. A parameter that greatly influences the nutritional profile of the elderly patient is the speed of chewing. Indeed, chewing too fast tends to increase the risk of overweight and obesity at all ages [16,17]. It has also been shown that chewing practiced with conscious effort is much more effective than chewing without it. In the elderly, it might be difficult to obtain the conscious effort of chewing because a cognitive impairment is often present. This is an important problem because the inadequate formation of a food bolus can easily cause suffocation and aspiration pneumonia due to the impaired swallowing function [18]. Other reasons why chewing is so important is that it activates the neurons of the histaminergic system, has beneficial effects on the diet reducing frequency of meals and benefits over the kinetics of digestion and the bioavailability of macronutrients [19]. Another fact to consider is that anorexia in the elderly is related to other eating patterns. Indeed, it is known that with advancing age, there is a progressive reduction in the daily caloric intake [20]. The consumption of foods with a high fat component is especially penalized. The importance of a diet with an adequate protein component for the well-being of the elderly is universally recognized, especially as regards bone and muscle health since the mass of these structures, fundamental both to maintaining homeostasis and in social behavior, tends to decrease significantly with aging [21,22,23]. On the other hand, fruit and vegetables are essential for health for many reasons: they reduce the risk of cognitive decline; they modulate inflammatory, oxidative phenomena and mineral density; they are also a precious source of micronutrients, including several vitamins, which are important for the performance of normal cellular functions [24,25,26,27,28,29,30,31,32].

Unfortunately, all studies present in the literature tend to focus the attention on young and adult populations. In view of this, the innovation of this study consists in having collected clinical data from very old patients (over 80 years of age). Therefore, the objective of this paper is detecting correlations between masticatory bite force (MBF) and body mass index (BMI) vs. age and/or sex in people with full natural dentition.

## 2. Materials and Methods

### 2.1. Study Design and Methodology

In the years 2018–2019, all patients of the dentist ambulatory in the Department of Geriatric Dentistry (Policlinico “Umberto I” University Hospital, Rome, Italy) were screened for study inclusion. A sample of university students and middle-aged nurses were also screened for inclusion.

The study protocol was organized respecting of 1975 Helsinki Declaration and the Ethical Committee of Sapienza University approved it (n. 09092020). After medical explanation, all patients read and signed a written informed consent.

### 2.2. Sample Size and Inclusion Criteria

The sample was composed of 426 subjects (213 females plus 213 male).

Inclusion criteria were as follows: the absence of psychiatric disorders; the absence of TMJ or masticatory muscles disfunction; the absence of spontaneous dental pain, angle dental malocclusions, periodontitis or pain induced by chewing.

Exclusion criteria were as follows: the presence of psychiatric disorders, TMJ disfunction, masticatory muscles disfunction, spontaneous dental pain, angle dental malocclusion, periodontitis (periodontal pocket > 5 mm) or pain induced by chewing.

All enrolled subjects were assigned into five different groups by age for minimizing bias within and between groups.

- Group “A” = aging from 20 to 35 years.
- Group “B” = aging from 45 to 59 years.
- Group “C” = aging from 60 to 69 years.
- Group “D” = aging from 70 to 79 years.
- Group “E” = aging more than 79 years.

All patients underwent physical and medical examinations. All enrolled subjects had no problems in everyday life and had a complete natural dentition, not considering the third molars.

Orthopantomography (OPG) image was used for evaluating periodontal conditions. We used OPG because it is a good mass screening method that requires low radiation and is time saving and low cost [33].

Using OPG we validated the number of teeth and the measure of dental bone retraction (in millimeters) for each tooth [34].

Maximum MBF was measured by a digital bite force dynamometer (KRATOS Equipamentos model IDDKv4, seral number 07175142. Sao Paulo, Brazil). For every measurement, the bite fork was covered with a new latex device to avoid contaminations and it was placed on first molar. After that, patients were asked to bite the fork as strongly as possible for 5 s. Bite force (kg) was recorded three time after intervals of two minutes [35].

### 2.3. Statistical Analysis

Data were collected into a Microsoft Access 2017 database, and they were analyzed by Epi-Info 2019 programs [CDC and NIH, 2019 version 7.2.3.1]. Statistical analysis estimated descriptive statistics, frequencies and significance in group differences. The statistical significance “between” groups was calculated on continuous variables. An analysis of variance (ANOVA) was performed to test the equality of means between groups for continuous variables, including Bonferroni and Newman–Keuls pairwise mean comparison tests. A Yates corrected Chi-square test was used for non-continuous variables by Statcalc and Analysis programs. A *p*-value less than 0.05 was considered significant, and 95% confidence intervals were also calculated.

## 3. Results

The study was performed on 426 subjects (213 females plus 213 male).

These results demonstrate that subjects were equally distributed by sex and age (as per protocol), as shown in Table 1.

Male subjects are on average taller and heavier than female subjects. However, the average body mass index did not show statistically significant differences in the average values with respect to sex, except in the subjects of group “A”, where males were more corpulent than female subjects (*p* < 0.05).

**Figure 1:**
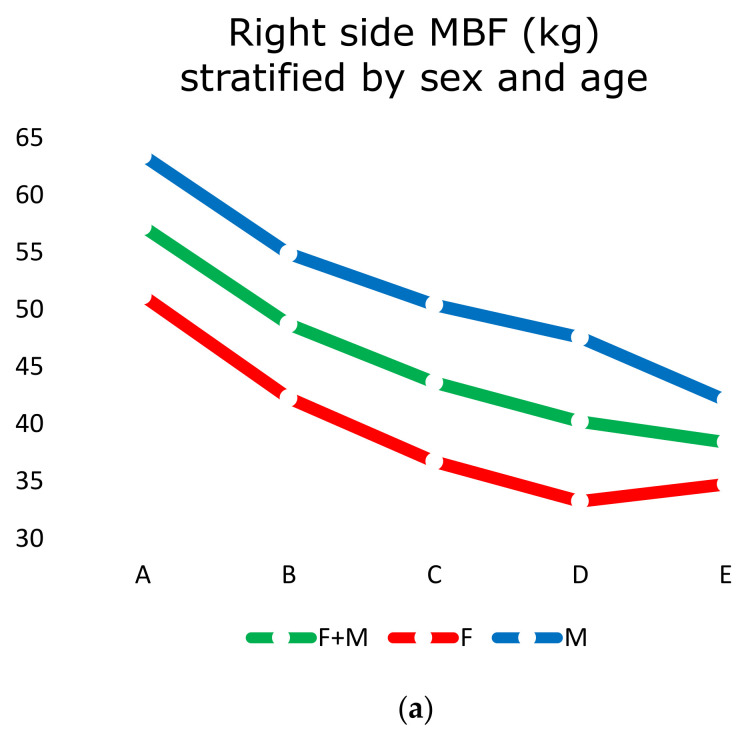

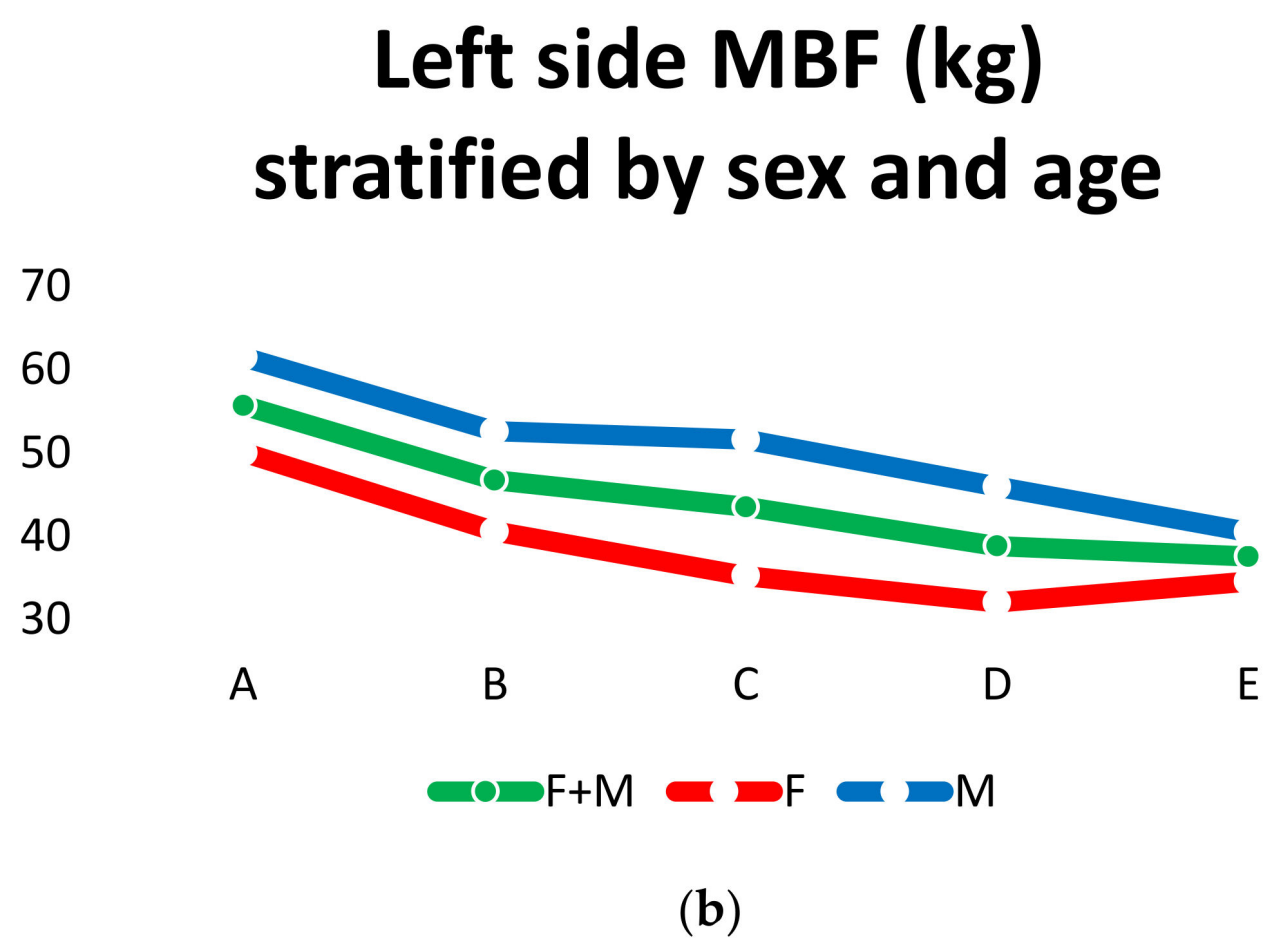
(**a**) Right side MBF (kg) stratified by sex and age; (**b**) left side MBF (kg) stratified by sex and age.

MBF (kg) was recorded on the right side (Table 2a and Figure 1a) and on the left side (Table 2b and Figure 1b) in all subjects.

There were not statistically significant differences in right-side MBF versus left-side MBF. The differences between sex were statistically significant with a stronger bite in males than females (Table 2a,b). At the same time, younger subjects had a stronger bite than older ones (*p* < 0.05; Figure 1a,b).

**Figure 2:**
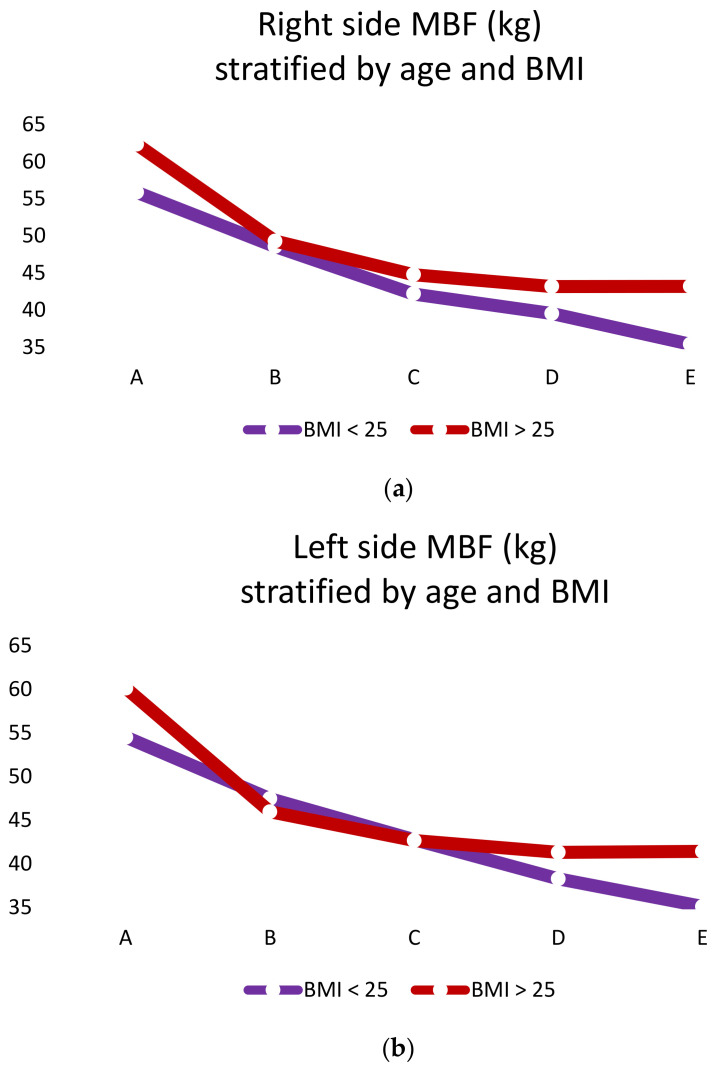
(**a**) Right side MBF (kg) stratified by age and BMI; (**b**) left side MBF(KG) stratified by age and BMI.

The analysis of mean MBF did not show statistically significant differences between groups “A”, “B”, “C” and “D” with subjects stratified by BMI. On the contrary, group “E” subjects showed a statistically significant difference correlated to BMI. In fact, more corpulent subjects (BMI > 25) had a MBF higher than less corpulent subjects (BMI < 25, *p* < 0.05; Figure 2a,b).

The analysis of mean MBF showed a statistically significant difference within all groups stratified by BMI with mean values inversely proportional with age (*p* < 0.001)

## 4. Discussion

The results in this study confirm the data from many scientific papers [1,4,12,36,37,38]. The aim of this paper is to correlate data between and within a large sample with a wide range of ages. Differences in the results of this paper and other papers are probably due to the smaller and younger samples used in previous research [1,39,40].

MBF is an important factor to evaluate functional status of masticatory system. A high mean MBF was found in each class of age. These data indicate an excellent masticatory performance in subjects who have all permanent teeth, despite increasing age. In fact, group “E” subjects (aged more than 79) showed a mean MBF of 35 kg in female and 42 kg in male.

The MBF values decrease with age. The more significant MBF decrease was 8–9 kg, which was present from group “A” (aged 25–35) to group “B” (aged 45–59). Considering MBF mean values vs. age, females showed lower data but had less of a decrease than males.

The MBF decreases with increasing age. This fact may be due to physiologic muscle involution, particularly in masticatory muscular mass. These muscular mass involutions promote loss of speed and precision in contraction in masticatory movements in elders [1,11,12,41].

Valid alimentation is not only connected with masticatory action, but also with the capability of penetrating different kinds of food. The literature shows the different cutting forces needed for rye bread (167 N—17 kg), raw carrot (118 N—12 kg), boiled meat (80 N—8 kg), raw cabbage (74 N—7 kg) and cooked meat (124 N—13 kg) [1,40,42,43,44].

MBF is a voluntary effort requested of the patients that only lasts for 5 s, but lower forces are used during normal mastication. Therefore, the MBF value cannot be considered the real habitual masticatory force used to grind and swallow food.

Gibbs CH et al. (1981) [45] demonstrated that mean bite force in dentate subjects during mastication is about 40% of their MBF. The subjects of our study had a mean bite force well above 8 kg, which is the force necessary for chewing boiled meat. People in group “E” (aged more the 80) had a lower value in MBF than other younger subjects; also, our elders had the capability to eat a large variety of foods for a healthy and complete alimentation.

Previous papers showed that people with compromised dentition, impaired posterior tooth units or with partial/complete dentures have more of a decrease in MBF than complete dentate subjects due to less-trained jaw muscles [46].

The decreased functional demands cause a reduction and atrophy in type II fibers attributable to the altered functional demands [1,47,48]

Regarding BMI, in the literature, AL-Omiri et al. [49], as well as other authors [50,51], did not show a significant effect of BMI on MBF. A possible explanation for this could be the effect of physical training of certain muscle groups in adults due to sports or work [52], but also the use of smaller samples compared to the one in the current study.

Abu Alhaija et al. [53] reported a statistically significant positive correlation between body mass index (BMI) and bite force. In our study, the mean BMI showed a statistically significant difference between subjects’ genders only in group “A”, where men were more corpulent than women. There were not statistically significant differences in BMI correlated with MBF in people aged under 80. On the contrary, in group “E” subjects (aged over 80), people with BMI > 25 showed a MBF greater than people with BMI < 25 (*p* < 0.05). As in the literature there was no difference in the correlation made for age and BMI and MBF, if would be interesting to investigate when the correlation is significant and whether it is age-related as well.

## 5. Conclusions

In conclusion, even if MBF decreases with age, this does not justify malnutrition in the elderly because there are other factors to consider when evaluating chewing capability. Completeness of natural dentition, frailty and comorbidities should be analyzed item by item in further studies to better understand how malnutrition is influenced by MBF. In view of these considerations, further experimental exploration in future works is needed.

Furthermore, of all the studies present in the literature, the attention has mostly been focused on young and adult populations. In view of this, the innovation of this study consists in having collected clinical data from a wider age range of patients, which included very old ones (over 80 years of age), therefore giving a broader perspective on the matter.

## Figures and Tables

**Table 1:** Anthropometric measures stratified by sex and age.

Age Group	“A” 20~35 Years Old	“B” 45~59 Years Old	“C” 60~69 Years Old	“D” 70~79 Years Old	“E” 80~99 Years Old	
Sex	40 F + 39 M	43 F + 45 M	48 F + 49 M	42 F + 40 M	40 F + 40 M	
Mean Age (years ± SD)	25 ± 5	52 ± 5	64 ± 3	75 ± 3	86 ± 3	*p* < 0.0001
F—Height (cm)	167 ± 6	163 ± 6	163 ± 6	160 ± 5	159 ± 5	*p* < 0.0001
M—Height (cm)	175 ± 8	173 ± 8	171 ± 7	169 ± 7	169 ± 6	
F—Weight (kg)	59 ± 10	66 ± 10	66 ± 8	64 ± 8	60 ± 9	*p* < 0.0001
M—Weight (kg)	73 ± 10	77 ± 15	75 ± 11	74 ± 10	70 ± 7	
F—BMI	21 ± 3 *	25 ± 3	25 ± 3	25 ± 3	24 ± 3	*p* < 0.001 *
M—BMI	24 ± 2 *	25 ± 3	26 ± 3	26 ± 3	24 ± 2	

* BMI differences in group “A” patients after stratitification per sex.

**Table 2:** (**a**) Right side MBF measures (kg) stratified by sex and age. (**b**) Left side MBF measures (kg) stratified by sex and age.

**(a)**						
**Age Group**	**Sex**	**Mean**	**D.S.**	**Median**	**Mode**	
“A”	40 F	**51**	**±16**	55	70	*p* < 0.01
	39 M	**63**	**±20**	65	29	
“B”	43 F	**42**	**±13**	40	52	*p* < 0.01
	45 M	**55**	**±23**	52	40	
“C”	48 F	**37**	**±12**	34	30	*p* < 0.001
	49 M	**50**	**±19**	48	39	
“D”	42 F	**33**	**±11**	33	35	*p* < 0.01
	40 M	**48**	**±20**	49	55	
“E”	40 F	**35**	**±12**	34	44	*p* < 0.05
	40 M	**42**	**±13**	40	26	
**(b)**						
**Age Group**	**Sex**	**Mean**	**D.S.**	**Median**	**Mode**	
“A”	40 F	**50**	**±17**	51	60	*p* < 0.05
	39 M	**61**	**±20**	61	75	
“B”	43 F	**40**	**±13**	40	35	*p* < 0.05
	45 M	**53**	**±23**	49	49	
“C”	48 F	**35**	**±14**	35	35	*p* < 0.0001
	49 M	**52**	**±20**	49	45	
“D”	42 F	**32**	**±11**	31	30	*p* < 0.01
	40 M	**46**	**±20**	48	50	
“E”	40 F	**35**	**±12**	32	25	*p* < 0.05
	40 M	**40**	**±14**	40	30	

## Data Availability

Data and methods used in the research were presented in sufficient. detail in the paper so that other researchers can replicate the work. Raw data must be publicly available.
